# Effects of climate change on niche shifts of *Pseudotrapelus dhofarensis* and *Pseudotrapelus jensvindumi* (Reptilia: Agamidae) in Western Asia

**DOI:** 10.1371/journal.pone.0197884

**Published:** 2018-05-30

**Authors:** Iman Rounaghi, Seyyed Saeed Hosseinian Yousefkhani

**Affiliations:** Young Researchers and Elite Club, Islamic Azad University, Shirvan branch, Shirvan, Iran; Universita degli Studi di Napoli Federico II, ITALY

## Abstract

Genus *Pseudotrapelus* has a wide distribution in North Africa and in the Middle East. In the present study, we modeled the habitat suitability of two Omani species of the genus (*Pseudotrapelus dhofarensis* and *Pseudotrapelus jensvindumi*) to evaluate the potential effects of climate change on their distribution. Mean diurnal range and precipitation of wettest quarter are the most highly contributed variables for *P*. *jensvindumi* and *P*. *dhofarensis*, respectively. The potential distribution for *P*. *dhofarensis* in the current time covers the southern coastal regions of Oman, Yemen, the Horn of Africa, and Socotra Island, but the suitable regions were reduced in the future prediction and limited to Yemen, Socotra Island, and Oman. There have not been any records of the species outside of Oman. Analysis of habitat suitability for *P*. *jensvindumi* indicated that the species is restricted to the Al Hajar Mountain of Oman and the southeast coastal region of Iran, but there are no records of the species from Iran. Because mean diurnal range will not be influenced by climate change in future, the potential distribution of the species is not expected to be changed in 2050. All predicted models were performed with the highest AUC (more than 0.97) using the Maxent method. Investigation to find unknown populations of these two species in Iran, Yemen, and Socotra Island is essential for developing conservation programs in the future.

## Introduction

Climate change and increasing human activities are increasingly affecting biodiversity around the world [1]. Climate change can cause species to change their distribution patterns according to the availability of suitable habitat regions [2], but may also change distributions by affecting other factors, such as predators and competitors. Therefore, future species distribution is related to a variety of biotic and abiotic factors. Additionally, habitat destruction and the degradation of ecosystems are important factors influencing species migration [3]. Species with small distribution ranges can be greatly affected by these changes, so it is important to estimate the effects of climate change in developing effective conservation programs for them. Biodiversity has been threatened by climate change and global warming effects [4] because these are important factors controlling the species expansion and dispersion [5]. Climate change can affect the amount of food resources for animals and, in addition to increasing temperature, can increase drought conditions [6].

The genus *Pseudotrapelus* has evolved around the Red Sea and is currently known from Arabia and Africa [7]. It was known as a monotypic genus (*Pseudotrapelus sinaitus*) for several years [8], but is now considered a species complex. Recently, morphological examination of different populations of the species showed that it contains at least five or six species within the complex [9]. Tamar et al. (2016) [7] revised the genus using different molecular markers and found that the genus contains different species because the previous studies focused on using only COI sequences. Based on Tamar et al. (2016) [7], six species of the genus occurred around the Red Sea and Arabia. Two recently described species of the genus *Pseudotrapelus* that are distributed in Oman (*P*. *dhofarensis*, *P*. *jensvindumi*) [10,11] have not been well-studied to understand their ecological features.

Ecological niche differentiation is one of the key criteria for species distinction [12,13]. The Maximum Entropy algorithm (MaxEnt), which uses only presence records and environmental layers, is a popular method for predicting species distribution [14, 15]. Ecological niche modeling (ENM) can be used for diverse aims such as species conservation [13,14,16], species recognition using ecological criteria [17, 18, 19], and the evaluation of the effects of global change on future species distributions [20, 21]. Species distribution modeling (SDM) is a method to predict the current conditions determining a species distribution range and to link recent climate change to the future distribution of a species [22]. For conservation purposes, it is important to verify species distribution using current presence records and to check different scenarios to find the best ways to protect these amazing creatures.

In the present study, we used presence records of both *Pseudotrapleus* species in Oman to achieve the following aims: 1) to predict the habitat suitability for these species in the southern Arabian Peninsula; and 2) to compare the future (2050) distribution prediction with current distribution pattern.

## Materials and methods

### Occurrence records and environmental data

**Fig 1:**
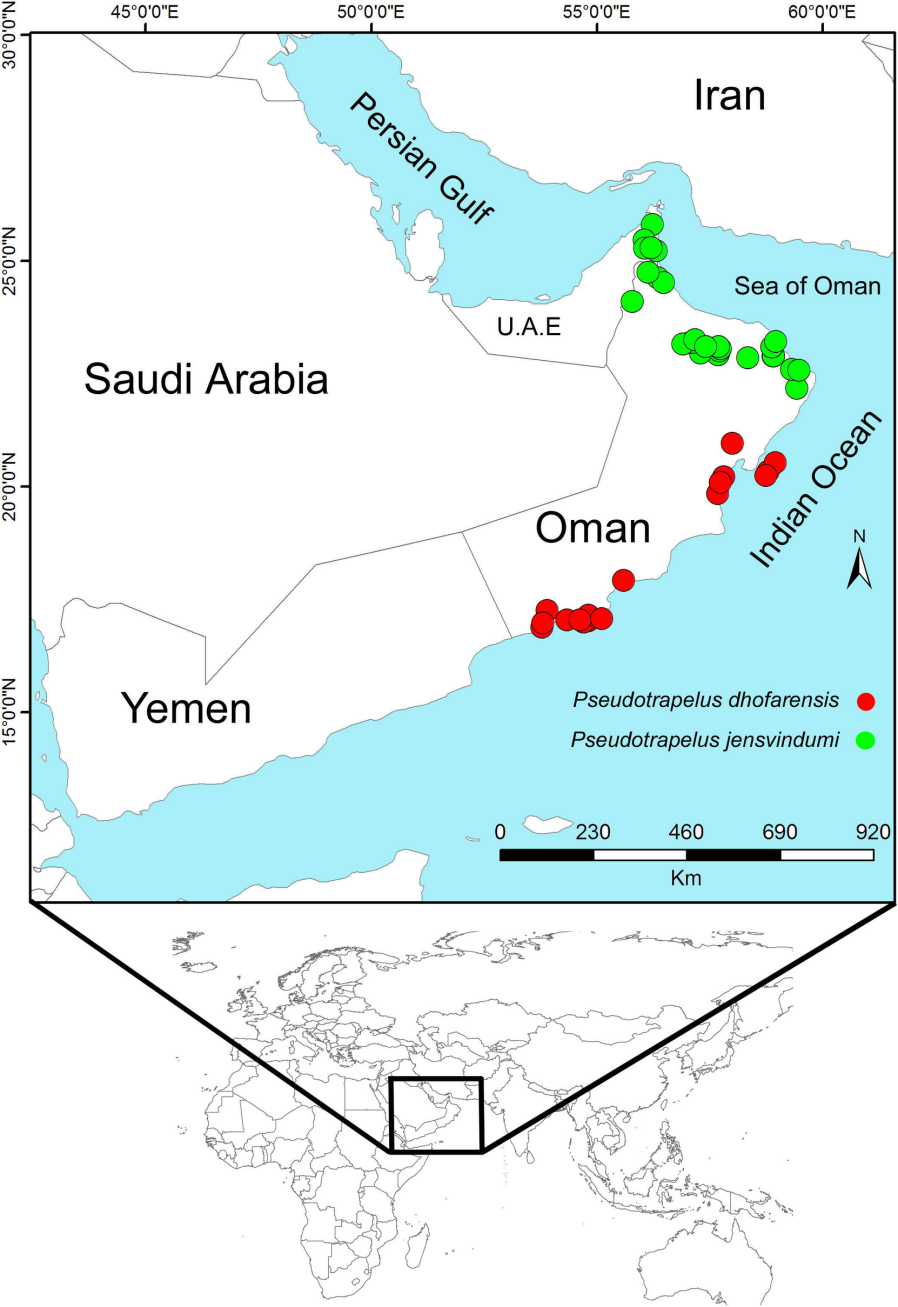
Distribution of Pseudotrapelus dhofarensis and P. jensvindumi in Oman.

Species presence records were obtained from previously published literature (Tamar et al., 2016) [7] and are presented as S1 Table (19 unique records for *P*. *dhofarensis* and 30 unique records for *P*. *jensvindumi*) (Fig 1). Nineteen bioclimatic layers for the current period (1950–2010) were downloaded from www.worldclim.org and 19 future bioclimatic layers were downloaded under recent IPCC reports for 2050 as four scenarios (2.6, 4.5, 6.0 and 8.5) from www.ccafs-climate.org (S2 Table). All layers were downloaded in 30 arc-sec resolution and clipped using ArcGIS 10.3 (ESRI) for the region of the Arabian Peninsula and adjacent countries. To avoid the effect of high correlation among layers, we examined all 19 layers using Openmodeller 1.0.7 [23] and then used the Pearson correlation method to obtain higher correlative layers (> 0.7). Layers with lower correlation (< 0.7) were selected for the analyses as follows: *Pseudotrapelus dhofarensis*: BIO4, BIO6, BIO15, BIO17, BIO18 and BIO19; *P*. *jensvindumi*: BIO2, BIO4, BIO17 and BIO19.

### Species distribution modeling

Species distribution was modeled for different time periods using MaxEnt 3.3.3e [24, 25, 26]. MaxEnt predicts the current distribution range and projects it to make a future prediction. Twenty-five percent of all data were considered as test data and the rest were used as training data. All setting parameters were left as default (regularization multiplier = 1; max number of background points = 10000) and the analysis was run using ten replicates as cross-validate type. Model accuracy was evaluated using area under the receiver-operating-characteristic curve (AUC), ranging between 0.5 and 1. The AUC of 1 means that the predicted model is very good [27]. The predicted models were run for the present time and then projected for 2050. All obtained models were evaluated using ENMTools [28] to find the percentage of niche overlap between current and future predicted models and to show the degree of change in suitable regions. For this aim, two values are important as Schoener’s *D* [28] and Hellinger’s-based *I* [Schoener 1968] that are two indices for niche overlap and were calculated based on the habitat suitability comparison from ENM.

## Results

**Fig 2:**
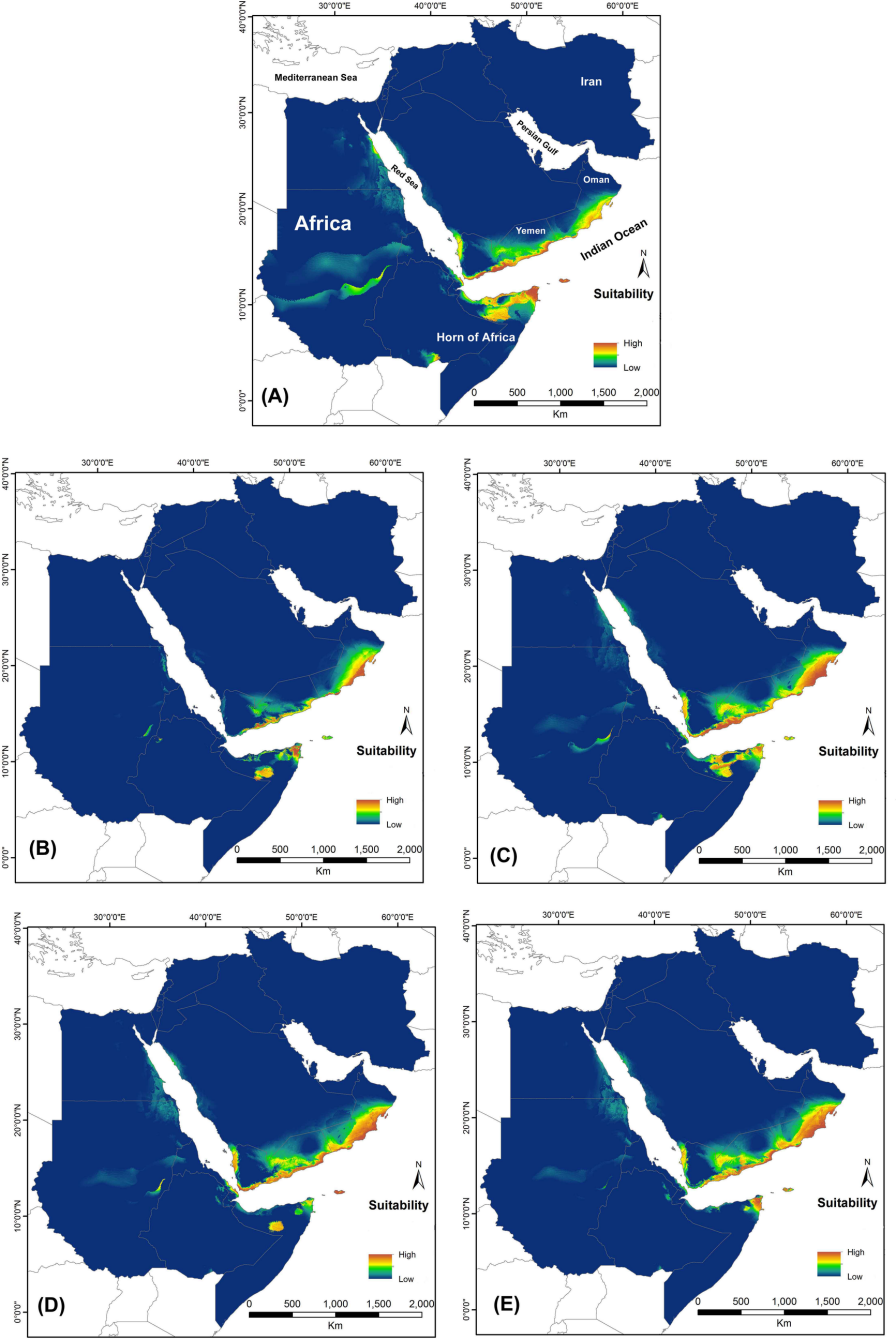
Potential distribution of *Pseudotrapelus dhofarensis* in A) the current period and in future periods under different scenarios as B) 2.6, C) 4.5, D) 6.0, and E) 8.5.

**Fig 3:**
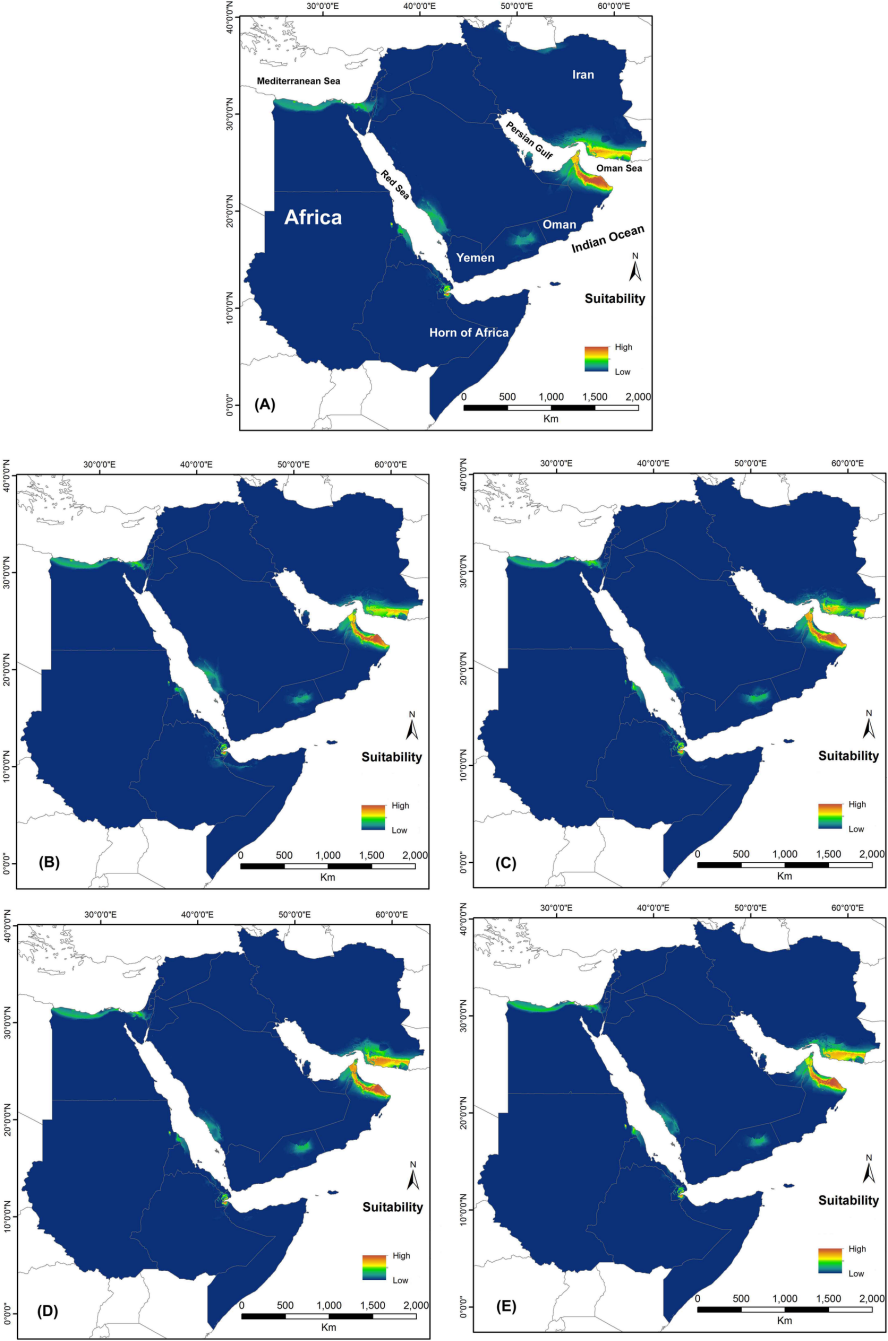
Potential distribution of *Pseudotrapelus jensvindumi* in A) the current period and in future periods under different scenarios as B) 2.6, C) 4.5, D) 6.0, and E) 8.5.

All models were run in ten replicates for two periods of time (the current time and future time of 2050) and the AUC values of all models were obtained with high accuracy values (Table 1). Contribution performance of layers for each period and scenarios are presented in Table 2. Based on the AUC values, the maps were predicted as very good level (more than 0.950). According to the result, BIO18 (mean diurnal range) and BIO2 (precipitation of warmest quarter) are the most contributed variables in the current habitat suitability predictions of *P*. *dhofarensis* and *P*. *jensvindumi*, respectively. However, the most contributed variables for the future period are BIO17, 4, and 2 in different scenarios (Table 2). Suitability of habitat for both species changed from the current period to the future time (Figs 2 and 3). The suitable area of *Pseudotrapelus dhofarensis* changed distinctly in the future prediction and was restricted to Oman and some coastal regions in Yemen; the species range in the Horn of Africa was reduced because of unsuitable areas (Fig 2). On the other hand, climate change is not predicted to affect the suitable habitat of *Pseudotrapelus jensvindumi* and future projections in different scenarios didn’t show any distribution differences between current and future periods. Southeastern Iran (Makran region) and the Hajar Mountains of Oman are the most favorable areas for the presence of *Pseudotrapelus jensvindumi*. Based on the niche overlap analysis, habitat suitability among current and future scenarios are overlapped more than 80% based on both Schoener’s *D* and Hellinger’s-based *I* values (S3 and S4 Tables).

**Table 1:** AUC values of all of the models that were performed on two species of the genus *Pseudotrapelus* in Oman.

Period	AUC±SD value (*P*. *dhofarensisi*)	AUC±SD value (*P*.*jensvindumi*)
Current	0.986±0.006	0.992±0.004
2.6_future (2050)	0.994±0.002	0.995±0.005
4.5_future (2050)	0.987±0.008	0.995±0.004
6.0_future (2050)	0.982±0.008	0.991±0.007
8.5_future (2050)	0.985±0.007	0.985±0.002

**Table 2:** All contributed variables (in percentages) for habitat suitability predictions for two *Pseudotrapelus* species. Bold numbers refer to the highly contributed variable in the model.

Bioclimatic layer	*P*. *dhofarensis*					*P*. *jensvindumi*				
	current	2.6	4.5	6.0	8.5	current	2.6	4.5	6.0	8.5
BIO 2	-	-	-	-	-	**39.8**	**37.1**	**37.1**	**37.1**	**37.1**
BIO 4	23.6	9	**35.8**	**35.8**	**35.8**	23.6	25.5	25.5	25.5	25.5
BIO 6	5.7	10.5	8.2	8.2	8.2	-	-	-	-	-
BIO 15	11.1	0.7	11.2	11.2	11.2	-	-	-	-	-
BIO 17	0	**30.2**	0	0	0	9.6	9.7	9.7	9.7	9.7
BIO 18	**44.5**	25.4	31.3	31.3	31.3	-	-	-	-	-
BIO 19	15.1	24.1	13.6	13.6	13.6	27	27.7	27.7	27.7	27.7

## Discussion

The current distribution patterns of both agama species from Western Asia is restricted to Oman (Fig 1) [7]. The predicted models for both species show a wide suitable range of habitat in the Horn of Africa, Yemen, and south eastern Iran (Figs 2 and 3), mostly affected by precipitation in the wettest quarter and mean diurnal range. Based on Tamar et al. (2016) [7], both of these species have been present for more than 5 million years in Arabia, so we can consider them as ancient species. With this long history, it is assumed that they may be present in Yemen and Iran, but, as with some other reptile species, there may be deficient data. *Pseudotrapleus jensvindumi* has a very limited distribution range and is restricted to Oman and the region around the Al Hajar Mountains [11]. *Pseudotrapelus dhofarensis* also has a small distribution range in western and southern Oman. Based on the Melnikov and Melnikova (2013) [11] and Tamar et al. (2016) [7], we cannot confirm the presence of this species in Yemen.

The potential distribution areas of these species have not been previously predicted using modeling approaches, but we have modeled them using the Maxent approach and 7 bioclimatic layers. Our results indicate that some bioclimatic layers that explain the current distribution pattern of the species may not affect the species distribution for future. For example, *P*. *jensvindumi* has a known distribution in the northern part of Oman along the Al Hajar Mountain [11] and the most effective layer is the mean diurnal range of the year. This variable is not influenced by climate change, so we may expect that the distribution range of this species will not change from the present (Fig 3). The second effective layer is BIO19 (precipitation of coldest quarter). The most probable value of the precipitation reported is about 100 mm and a value higher than this has a negative effect on the species presence [29]. Based on the current prediction, this region normally has low precipitation and therefore climate change is not predicted to directly affect suitable habitats in the future. Based on the niche overlap analysis, habitat suitability among current and future different scenarios overlapped more than 80% and confirms that climate change cannot affect the distribution pattern of the species in future (S4 Table). The suitability of habitat for *P*. *jensvindumi* on both sides of Oman Sea can tell us about the species presence in southeastern Iran because the Persian Gulf dried several times during the Pleistocene period [30] and several taxa entered into the Iranian Plateau, such as *Pseudoceramodactylus khobarensis* [31] and *Pristorus rupestris* [32]. If *P*. *jensvindumi* was represented in Iran, then this happened recently. However, we don’t have any records of the species in the region and there is a need for more investigation in southeast Iran.

Another species, *P*. *dhofarensis*, was described from the Dhofar region in southern Oman [11] and Tamar et al. (2016) [7] confirmed its specific status. We modeled the habitat suitability of this species and southern coastal regions of Oman, Yemen, Socotra Island, and the Horn of Africa were predicted as suitable areas. However, the models predicted that climate change in future and reductions in the amount of precipitation in the wettest quarter would restrict its potential distribution to Yemen, Socotra Island, and Oman (Fig 2). Reductions in suitable regions were evaluated by niche overlap and confirmed that the suitable region for *P*. *dhofarensis* could be changed by 15–20% (S3 Table). This contraction of suitable habitat may be affected by change in vegetation type, so it is necessary to manage conservation programs regarding these species in Oman. Our predicted models for both species in the current and future periods indicated that these species can inhabit a wider area than is presently known. Habitat in southern Yemen, the Horn of Africa, and southeastern Iran show the widest suitable range for the presence of both species (Figs 2 and 3). So far, different ecological studies have been done on the effects of climate change on the predictions of habitat suitability [33, 34] for reptiles and mammals. They referred to decreasing amounts of suitable habitats for different species related to changes in precipitation and temperature, representing the two main global challenges faced by these animals. Human activitiesare the main cause for decreasing the suitable habitats in some animals, but most reptiles avoid densely populated regions and therefore their suitable habitats are influenced more by temperature and precipitation. Generally, because of their high dispersal ability, these two species can change their distribution range as climate changes, especially *Pseudotrapelus dhofarensis* from the Horn of Africa to south Yemen.

In conclusion, by comparing both current and predicted future maps, we can suggest that a conservation program is needed to evaluate *P*. *dhofarensis* because of predicted reduction in suitable habitat regions in future, but that *P*. *jensvindumi* is not likely to be at major risk.

## Supporting information

S1 Table List of records and coordinates from Oman used in this study. (DOCX)

S2 Table Bioclimatic variables used in the models in this study. (DOCX)

S3 Table Schoener’s *D* (above diangonal) and Hellinger’s-based *I* (below diagonal) values from niche overlap of *Pseudotrapelus dhofarensis*. (DOCX)

S4 Table Schoener’s *D* (above diangonal) and Hellinger’s-based *I* (below diagonal) values from niche overlap of *Pseudotrapelus jensvindumi*. (DOCX)
