# Supplementary material for: Effects of climate change on niche shifts of Pseudotrapelus dhofarensis and Pseudotrapelus jensvindumi (Reptilia: Agamidae) in Western Asia
Source: PLoS One. 2018 May 30;13(5):e0197884. doi: 10.1371/journal.pone.0197884 (PMC5976179; doi:10.1371/journal.pone.0197884)
Supplement: S1 Table — (DOCX) [file pone.0197884.s001.docx]

**S1 Table.** List of records and coordinates from Oman used in this study.

| Species | Latitude | Longitude |
| --- | --- | --- |
| *Pseudotrapelus dhofarensis* | 16.8793 | 53.7743 |
| *Pseudotrapelus dhofarensis* | 16.9774 | 53.7919 |
| *Pseudotrapelus dhofarensis* | 16.9942 | 54.6912 |
| *Pseudotrapelus dhofarensis* | 17.0244 | 54.7983 |
| *Pseudotrapelus dhofarensis* | 17.0413 | 54.326 |
| *Pseudotrapelus dhofarensis* | 17.0413 | 54.611 |
| *Pseudotrapelus dhofarensis* | 17.0693 | 55.0936 |
| *Pseudotrapelus dhofarensis* | 17.0708 | 55.093 |
| *Pseudotrapelus dhofarensis* | 17.1482 | 54.7951 |
| *Pseudotrapelus dhofarensis* | 17.2497 | 53.8891 |
| *Pseudotrapelus dhofarensis* | 17.9172 | 55.5766 |
| *Pseudotrapelus dhofarensis* | 19.84 | 57.6639 |
| *Pseudotrapelus dhofarensis* | 20.0843 | 57.722 |
| *Pseudotrapelus dhofarensis* | 20.2098 | 57.7976 |
| *Pseudotrapelus dhofarensis* | 20.21 | 57.7971 |
| *Pseudotrapelus dhofarensis* | 20.2398 | 58.7293 |
| *Pseudotrapelus dhofarensis* | 20.3349 | 58.7885 |
| *Pseudotrapelus dhofarensis* | 20.5215 | 58.9415 |
| *Pseudotrapelus dhofarensis* | 20.9501 | 57.9832 |
| *Pseudotrapelus jensvindumi* | 22.1691 | 59.4135 |
| *Pseudotrapelus jensvindumi* | 22.5747 | 59.4646 |
| *Pseudotrapelus jensvindumi* | 22.592 | 59.3011 |
| *Pseudotrapelus jensvindumi* | 22.8476 | 58.3287 |
| *Pseudotrapelus jensvindumi* | 22.8855 | 58.904 |
| *Pseudotrapelus jensvindumi* | 22.8892 | 58.8929 |
| *Pseudotrapelus jensvindumi* | 22.9116 | 57.6746 |
| *Pseudotrapelus jensvindumi* | 22.948 | 57.2847 |
| *Pseudotrapelus jensvindumi* | 23.0026 | 57.7001 |
| *Pseudotrapelus jensvindumi* | 23.051 | 57.7242 |
| *Pseudotrapelus jensvindumi* | 23.0854 | 58.8673 |
| *Pseudotrapelus jensvindumi* | 23.086 | 58.8673 |
| *Pseudotrapelus jensvindumi* | 23.09 | 57.6826 |
| *Pseudotrapelus jensvindumi* | 23.0968 | 57.4001 |
| *Pseudotrapelus jensvindumi* | 23.1499 | 56.8937 |
| *Pseudotrapelus jensvindumi* | 23.1513 | 57.1918 |
| *Pseudotrapelus jensvindumi* | 23.1841 | 57.0426 |
| *Pseudotrapelus jensvindumi* | 23.1849 | 57.1412 |
| *Pseudotrapelus jensvindumi* | 23.2052 | 57.1457 |
| *Pseudotrapelus jensvindumi* | 23.2084 | 58.9458 |
| *Pseudotrapelus jensvindumi* | 23.2484 | 57.163 |
| *Pseudotrapelus jensvindumi* | 24.0966 | 55.7672 |
| *Pseudotrapelus jensvindumi* | 24.5152 | 56.4622 |
| *Pseudotrapelus jensvindumi* | 24.6203 | 56.34 |
| *Pseudotrapelus jensvindumi* | 24.7361 | 56.1264 |
| *Pseudotrapelus jensvindumi* | 25.2166 | 56.3 |
| *Pseudotrapelus jensvindumi* | 25.2711 | 56.0437 |
| *Pseudotrapelus jensvindumi* | 25.2805 | 56.1863 |
| *Pseudotrapelus jensvindumi* | 25.453 | 56.0324 |
| *Pseudotrapelus jensvindumi* | 25.795 | 56.2205 |
