# Supplementary material for: Effects of climate change on niche shifts of Pseudotrapelus dhofarensis and Pseudotrapelus jensvindumi (Reptilia: Agamidae) in Western Asia
Source: PLoS One. 2018 May 30;13(5):e0197884. doi: 10.1371/journal.pone.0197884 (PMC5976179; doi:10.1371/journal.pone.0197884)
Supplement: S2 Table — (DOCX) [file pone.0197884.s002.docx]

**S2 Table.** Bioclimatic variables used in the models in this study.

| **Variable** | **Definition** |
| --- | --- |
| BIO1 | Annual Mean Temperature |
| BIO2 | Mean Diurnal Range [Mean of monthly (max temp - min temp)] |
| BIO3 | Isothermality [(var2 / var7) * 100] |
| BIO4 | Temperature Seasonality (standard deviation * 100) |
| BIO5 | Maximum Temperature of Warmest Month |
| BIO6 | Minimum Temperature of Coldest Month |
| BIO7 | Temperature Annual Range (var5 - var6) |
| BIO8 | Mean Temperature of Wettest Quarter |
| BIO9 | Mean Temperature of Driest Quarter |
| BIO10 | Mean Temperature of Warmest Quarter |
| BIO11 | Mean Temperature of Coldest Quarter |
| BIO12 | Annual Precipitation |
| BIO13 | Precipitation of Wettest Month |
| BIO14 | Precipitation of Driest Month |
| BIO15 | Precipitation Seasonality (standard deviation / mean) |
| BIO16 | Precipitation of Wettest Quarter |
| BIO17 | Precipitation of Driest Quarter |
| BIO18 | Precipitation of Warmest Quarter |
| BIO19 | Precipitation of Coldest Quarter |
