# Supplementary material for: Effects of climate change on niche shifts of Pseudotrapelus dhofarensis and Pseudotrapelus jensvindumi (Reptilia: Agamidae) in Western Asia
Source: PLoS One. 2018 May 30;13(5):e0197884. doi: 10.1371/journal.pone.0197884 (PMC5976179; doi:10.1371/journal.pone.0197884)
Supplement: S3 Table — (DOCX) [file pone.0197884.s003.docx]

**S3 Table.** Schoener’s *D* (above diangonal) and Hellinger’s-based *I* (below diagonal) values from niche overlap of *Pseudotrapelus dhofarensis*.

| Species | 1 | 2 | 3 | 4 | 5 |
| --- | --- | --- | --- | --- | --- |
| 1) Current |  | 0.99 | 0.99 | 0.99 | 0.99 |
| 2) Future_2.6 | 0.99 |  | 0.66 | 0.65 | 0.66 |
| 3) Future _4.5 | 0.99 | 0.86 |  | 0.81 | 0.80 |
| 4) Future _6.0 | 0.99 | 0.86 | 0.93 |  | 0.85 |
| 5) Future _8.5 | 0.99 | 0.85 | 0.92 | 0.95 |  |
