# Supplementary material for: Effects of climate change on niche shifts of Pseudotrapelus dhofarensis and Pseudotrapelus jensvindumi (Reptilia: Agamidae) in Western Asia
Source: PLoS One. 2018 May 30;13(5):e0197884. doi: 10.1371/journal.pone.0197884 (PMC5976179; doi:10.1371/journal.pone.0197884)
Supplement: S4 Table — (DOCX) [file pone.0197884.s004.docx]

**S4 Table.** Schoener’s *D* (above diangonal) and Hellinger’s-based *I* (below diagonal) values from niche overlap of *Pseudotrapelus jensvindumi*.

| Species | 1 | 2 | 3 | 4 | 5 |
| --- | --- | --- | --- | --- | --- |
| 1) Current |  | 0.96 | 0.96 | 0.96 | 0.65 |
| 2) Future_2.6 | 0.96 |  | 0.85 | 0.88 | 0.88 |
| 3) Future _4.5 | 0.96 | 0.97 |  | 0.86 | 0.84 |
| 4) Future _6.0 | 0.96 | 0.97 | 0.97 |  | 0.90 |
| 5) Future _8.5 | 0.65 | 0.98 | 0.97 | 0.98 |  |
